# β-Arrestin-Dependent Deactivation of Mouse Melanopsin

**DOI:** 10.1371/journal.pone.0113138

**Published:** 2014-11-17

**Authors:** Evan G. Cameron, Phyllis R. Robinson

**Affiliations:** Department of Biological Sciences, University of Maryland, Baltimore County, Baltimore, Maryland, United States of America; Doheny Eye Institute and Keck School of Medicine of the University of Southern California, United States of America

## Abstract

In mammals, the expression of the unusual visual pigment, melanopsin, is restricted to a small subset of intrinsically photosensitive retinal ganglion cells (ipRGCs), whose signaling regulate numerous non-visual functions including sleep, circadian photoentrainment and pupillary constriction. IpRGCs exhibit attenuated electrical responses following sequential and prolonged light exposures indicative of an adaptational response. The molecular mechanisms underlying deactivation and adaptation in ipRGCs however, have yet to be fully elucidated. The role of melanopsin phosphorylation and β-arrestin binding in this adaptive process is suggested by the phosphorylation-dependent reduction of melanopsin signaling *in vitro* and the ubiquitous expression of β-arrestin in the retina. These observations, along with the conspicuous absence of visual arrestin in ipRGCs, suggest that a β-arrestin terminates melanopsin signaling. Here, we describe a light- and phosphorylation- dependent reduction in melanopsin signaling mediated by both β-arrestin 1 and β-arrestin 2. Using an *in vitro* calcium imaging assay, we demonstrate that increasing the cellular concentration of β-arrestin 1 and β-arrestin 2 significantly increases the rate of deactivation of light-activated melanopsin in HEK293 cells. Furthermore, we show that this response is dependent on melanopsin carboxyl-tail phosphorylation. Crosslinking and co-immunoprecipitation experiments confirm β-arrestin 1 and β-arrestin 2 bind to melanopsin in a light- and phosphorylation- dependent manner. These data are further supported by proximity ligation assays (PLA), which demonstrate a melanopsin/β-arrestin interaction in HEK293 cells and ipRGCs. Together, these results suggest that melanopsin signaling is terminated in a light- and phosphorylation-dependent manner through the binding of a β-arrestin within the retina.

## Introduction

G-protein coupled receptors (GPCRs) make up the largest super-family of integral membrane receptors found in almost all eukaryotic organisms [1], [2]. Upon activation, GPCRs typically couple to heterotrimeric G-proteins that regulate the activation of downstream effectors including adenylate cyclase (AC), phosphodiesterase (PDE), and phospholipase C (PLC). At the receptor level, GPCR signaling is regulated in a two-step manner that involves phosphorylation of the receptor carboxyl-tail (C-tail) followed by the binding of an arrestin. Generally, GPCR C-tail phosphorylation serves two purposes 1.) to attenuate receptor signaling and 2.) to promote the activation and binding of an arrestin. Once bound, arrestin completely quenches receptor signaling, and in some cases directs receptor internalization [3]–[8]. Additionally, GPCR-bound arrestins can serve as independent signal transducers that functionally oppose or prolong G-protein signaling [9]–[11].

Mammals express four isoforms of arrestin: arrestin 1 (rod arrestin), arrestin 2 (β-arrestin 1), arrestin 3 (β-arrestin 2) and arrestin 4 (cone arrestin). Rod and cone arrestin or “visual arrestins” are expressed exclusively in the photoreceptor layer of the retina where they terminate visual pigment signaling [12]. Conversely, β-arrestin 1 and β-arrestin 2 are ubiquitously expressed and generally regulate non-visual GPCR signaling. Functionally, all four arrestins deactivate GPCRs by binding to the intracellular loops of activated receptors and sterically blocking G-protein binding and activation. Additionally, β-arrestins can further attenuate receptor signaling by promoting receptor endocytosis. This process is facilitated by clathrin and adaptor-2 (AP2) binding domains in the C-tails of both β-arrestins that direct arrestin-bound receptor complexes to clathrin coated pits. These clathrin and AP2 binding motifs are absent in the visual arrestins, whose binding generally does not promote receptor internalization [13]–[17].

Opsin visual pigments make up a specialized class of GPCRs that detect light. Historically, visual pigments in the mammalian retina were thought to reside exclusively in the rod and cone photoreceptors. However, the discovery of the unusual visual pigment, melanopsin, in a small subset of retinal ganglion cells (RGCs) disproved this idea. Expression of melanopsin confers photosensitivity to RGCs which are referred to as intrinsically photosensitive retinal ganglion cells or ipRGCs. The axons of ipRGCs have been shown to primarily project to brain nuclei known to regulate circadian photoentrainment, pupillary light response, and sleep [18]–[24]. Unlike rhodopsin and cone opsins, melanopsin is thought to couple to a G_q_-mediated signaling pathway that results in a cellular depolarization. This response is akin to that mediated by rhabdomeric opsins (typically expressed in invertebrate photoreceptors) whose activation also culminates in a cellular depolarization [25]–[27]. Several lines of evidence suggest that ipRGCs signal continuously throughout the day, relaying gross luminance information to the brain [28]–[30]. Additionally, melanopsin-expressing cells have been shown to adapt in response to prolonged light exposures in retinal recordings and in isolated single cell recordings following short paired light pulses and series of flashes [28], [31], [32]. Collectively, these observations raise questions about how melanopsin signaling is regulated. Here we provide evidence for a light- and phosphorylation-dependent deactivation of melanopsin by β-arrestin in HEK293 cells and in the murine retina. Furthermore, we shed light on the importance of C-tail phosphorylation and β-arrestin binding on melanopsin adaptation and signaling.

## Materials and Methods

### Immunohistochemistry

Whole eye-cups from adult mice (C57BL/6 & opn4^LacZ/Mel^) were isolated and fixed in 4% formaldehyde (Thermo, Rockford, IL) for 12 hours. Following fixation, the cornea and lens were removed and each eye-cup was immersed in 30% glucose for approximately 2 hours at 4°C. Eye-cups were then frozen in OCT at −80°C, sectioned vertically on a cryostat to 16 µm and mounted on Superfrost Plus slides (Fisher Scientific, Pittsburg, PA). The Animal Care and Use Committee of University of Maryland, Baltimore County approved all animal care and procedures.

Mounted sections were blocked for 1 hour in 10% normal goat serum diluted in 0.3% Triton×100/1×PBS and probed with anti-melanopsin (N-terminal), anti-β-galactosidase (β-gal), anti-visual arrestin, anti-β-arrestin 1 and anti-β-arrestin 2 antibodies. Visual arrestin localization was detected using a rabbit polyclonal anti-visual arrestin antibody (1∶50) (Santa Cruz H-90). A chicken polyclonal anti-β-gal (1∶1500) (Aves, BGL-1040) and a rabbit monoclonal anti-β-arrestin 1 antibody (1∶100) (Epitomics, 1274-1) were used to show melanopsin/β-arrestin 1 co-localization. A custom rabbit polyclonal antibody specific for the N-terminus of mouse melanopsin (1∶1000) (Covance) was used in combination with a mouse monoclonal anti-β-arrestin 2 antibody (1∶100) (Sigma-Aldrich, WH0000409M1) to show melanopsin/β-arrestin 2 co-localization. Visual arrestin labeling was demonstrated using an Alexa-488 goat anti-rabbit antibody (1∶200) (Molecular Probes, A11011). Alexa-647 goat anti-chicken (1∶200) (Molecular Probes, A21449), Alexa-594 rabbit anti-mouse (1∶200) (Molecular Probes, A11062) and Alexa-488 goat anti-rabbit (1∶200) (Molecular Probes, A11011) were used to visualize anti-β-gal, anti-β-arrestin 1, anti-β-arrestin 2 and anti-melanopsin labeling, respectively. Nuclei were visualized with DAPI. Images were acquired using a Leica SP5 TCS Confocal microscope. Final processing including color balancing and contrast adjustments were performed in Image J (NIH freeware).

### Plasmids

Mouse melanopsin (Accession NP_038915) and mouse melanopsin lacking C-tail phosphorylation sites (phosphonull) were amplified by PCR using primers that included an EcoRI and SalI restriction sites at the 5′ and 3′ ends, respectively. The melanopsin stop codon was removed from the SalI 3′ primer to facilitate fusion with the 1D4 coding sequence of rhodopsin. 1D4 is a mouse monoclonal antibody whose epitope is made up of the last 9 amino acids of bovine rhodopsin [33]. The PCR products were digested and ligated into EcoRI/SalI digested pMT4 (pMT3 containing a synthetic bovine rhodopsin gene [34] to generate melanopsin-1D4 and phosphonull-1D4 expression constructs. Mouse melanopsin and phosphonull melanopsin pMT3 expression vectors lacking the 1D4 coding sequence were also generated. Rat β-arrestin 1 and mouse β-arrestin 2 pcDNA3.1 neo expression vectors were obtained from AddGene plasmid repository. All constructs were sequence verified (Genewiz).

### Generation of β-arrestin Stable Cell Lines

HEK293 cells used in this research were obtained from ATCC (Manassas, VA, no. CRL-1573). All cell lines were maintained in DMEM supplemented with 10% bovine serum and 1% penicillin/streptomycin. HEK293 cells were transfected with pcDNA-β-arrestin 1 or pcDNA-β-arrestin 2 using Turbofect (Thermo Scientific) as per the manufacturer's protocol. Transfected cells were selected with 1 mg/mL G418 (Sigma, St. Louis, MO) for 3 weeks and then screened. Stable β-arrestin over-expression was confirmed by immunoblotting compared to endogenous HEK293 β-arrestin levels.

### Heterologous expression and calcium imaging

HEK293 cells and β-arrestin stable cell lines were transfected with mouse melanopsin or phosphonull melanopsin expression constructs using Turbofect (Thermo Scientific) as per the manufacturer's protocol. Briefly, cells were counted and split into 6 well dishes at 350,000 cells per well and transfected the following day. 24 hour post transfection, the cells were released with 0.25% trypsin (Invitrogen, Carlsbad, CA), counted and re-plated in a clear bottom/black sided 96-well dish (BD, San Jose, CA) at a density of 7×10^4^ cells per well. The cells were then transferred to a dark room incubator to recover. All subsequent manipulations were carried out in the dark under dim red illumination. 24 hours later, cells were washed one time with Hank's balanced salt solution (HBSS) plus 20 mM HEPES, pH 7.4 and subsequently incubated in DMEM supplemented with 4 µM Fluo-4 (Molecular Probes, Eugene, OR), 0.02% pluronic acid (Invitrogen) and 20 µM 9-cis retinal (Sigma). The addition of 9-cis retinal in the dark is required for the reconstitution of the melanopsin visual pigment.

The melanopsin calcium response was measured on a Tecan Infinite M200 microplate reader (Tecan Group Ltd, Männedorf, Switzerland). A 485 nm excitation wavelength served to activate melanopsin and excite calcium bound Fluo-4, whose emission was detected at 520 nm. Excitation intensity was according to manufacturer's specifications and emission measurements were taken at a sampling rate of 1 Hz per 60 secs. All measurements were carried out in triplicate and baseline subtracted to account for background fluorescence.

### Crosslinking and co-immunoprecipitation

Five 10 cm plates of each β-arrestin stable cell line were transfected with pMT3 melanopsin-1D4 or phosphonull melanopsin-1D4 using Turbofect (Invitrogen). Post transfection, cells were immediately placed into a dark incubator for 48 hours to allow for protein expression. 48 hours later, cell media was replaced with 5 mL DMEM supplemented with 20 µM 9-cis retinal (Sigma) for 1 hour in the dark. After reconstitution, cells were exposed to bright white light in the presence of 500 ul of 25 mM dithiobis (succinimidyl propionate) (DSP; Pierce, Rockford, IL) for 30 mins at room temperature (20°C) with gentle rocking. DSP serves as a reversible crosslinker that “fixes” intracellular protein-protein interactions. DSP crosslinking was quenched by the addition of 50 mM Tris for 15 mins prior to cell harvesting. Following fixation, cells were washed 3 times with 1× PBS, harvested, flash frozen on dry ice and stored at −80°C.

Transfected cell pellets were solublized in 6 mL 1×PBS containing 1% dodecyl maltoside (DM; RPI, Mount Prospect, IL) and 1 mM phenylmethylsulfonylfluoride (PMSF; Sigma, St. Louis, MO) for 2 hours at 4°C. Solublized proteins were mixed with pre-bound 1D4 sepharose beads (GE Healthcare, Uppsala, SE) and incubated overnight at 4°C on a spinning carousel. Protein bound beads were spun down and washed 3 times with 1× PBS containing 0.1% DM and 1 mM PMSF. Proteins were eluted with 2 mL 1× PBS containing 50 mM DTT (Fisher Scientific) and incubated at 37°C on a spinning carousel for 1 hour. Elutions were concentrated to 100 ul with 2 mL 30 K Amicon Ultra centrifuge filters (Millipore, Billerica, MA), dissolved in laemmli sample buffer, and electrophoresed on a 15% SDS-polyacrylamide gel (150 V, 1 hr). Separated proteins were tank transferred to PVDF membranes (Millipore) in Tris/glycine buffer (100 V, 1 hr). Post transfer, PVDF membranes were incubated in 0.5% I-block (Applied Biosystems, Foster City, CA) in 10 mM Tris-Cl (pH 7.4), containing 0.1% Tween-80. Blots were probed with anti-1D4 (1∶5000) (Covance), anti-β-arrestin 1 (1∶1000) (Abcam, ab131180), and anti-β-arrestin 2 (1∶1000) (Sigma, WHOOOO409M1) antibodies and visualized using alkaline phosphatase-linked secondary antibodies and Attophos (Promega).

### Proximity Ligation Assay

β-arrestin 1 and β-arrestin 2 stable cell lines were transfected with mouse melanopsin and phosphonull melanopsin expression constructs using Turbofect (Thermo Scientific). Cells were counted and split into 6 well dishes at 350,000 cells per well and transfected the following day. 4 hours post transfection, the cells were released with 0.25% trypsin (Invitrogen) and re-plated in a new 6-well dish containing poly-L-lysine coated coverslips (BD Biosciences, Bedford, MA). Cells were then transferred to a dark room incubator to recover for 48 hours. Following recovery, transfected cells were incubated in 20 µM 9-cis retinal (Sigma) for 1 hour diluted in fresh DMEM. Cells were then fixed immediately in the dark with 4% PFA or exposed to 10 minutes of bright broad spectrum light (5000 lux) and then fixed. Following fixation, cells were washed 2 times with 1× PBS and mounted (cell side up) on microscope slides (Fisher Scientific, 12-550-15). For *in situ* PLA experiments, C57BL/6 and melanopsin knockout mice (opn4^LacZ/LacZ^) retinas were isolated as described previously for immunohistochemistry.

All proximity ligation assays (PLA) (Duolink, Olink Biosciences, CH) were carried out as previously described [35], [36]. Briefly, cells/tissue were blocked and permeabilized with Duolink 1× blocking solution for 1 hour. Samples were then probed with rabbit anti-melanopsin C-terminal antibody (1∶200) (Thermo Scientific, Cat #PA1-781) and mouse anti-β-arrestin 1 antibody (1∶200) (Abcam, ab167346) or mouse anti-β-arrestin 2 antibodies (1∶200) (Sigma, WH0000409M1) for 2 hours. Anti-mouse and anti-rabbit secondary antibodies conjugated to complimentary oligonucleotide strands (1∶5) (Olink, Cat#92002, Cat#92004) were used in conjunction with a Duolink detection kit (Olink, Cat#92007) to visualize the melanopsin/β-arrestin interaction.

The PLA assay permits detection of protein/protein interactions within fixed tissue or cells through the use of antibodies. Here we used primary antibodies raised against melanopsin, β-arrestin 1 and β-arrestin 2 to test their putative interaction in HEK293 cells and in the retina. In this assay, when a pair of primary antibodies comes within close enough proximity (<40 nm), they can be detected by secondary antibodies conjugated to oligonucleotides that will form a circular piece of DNA. This piece of DNA can then be amplified by rolling circle amplification to generate a concatemer of the template. Complimentary fluorescent oligonucleotide probes are then added to detect the amplified template and can be visualized as a bright fluorescent spot under a microscope.

## Results

### Melanopsin co-localizes with β-arrestin 1 and β-arrestin 2 in ipRGCs and not visual arrestin

**Figure 1:**
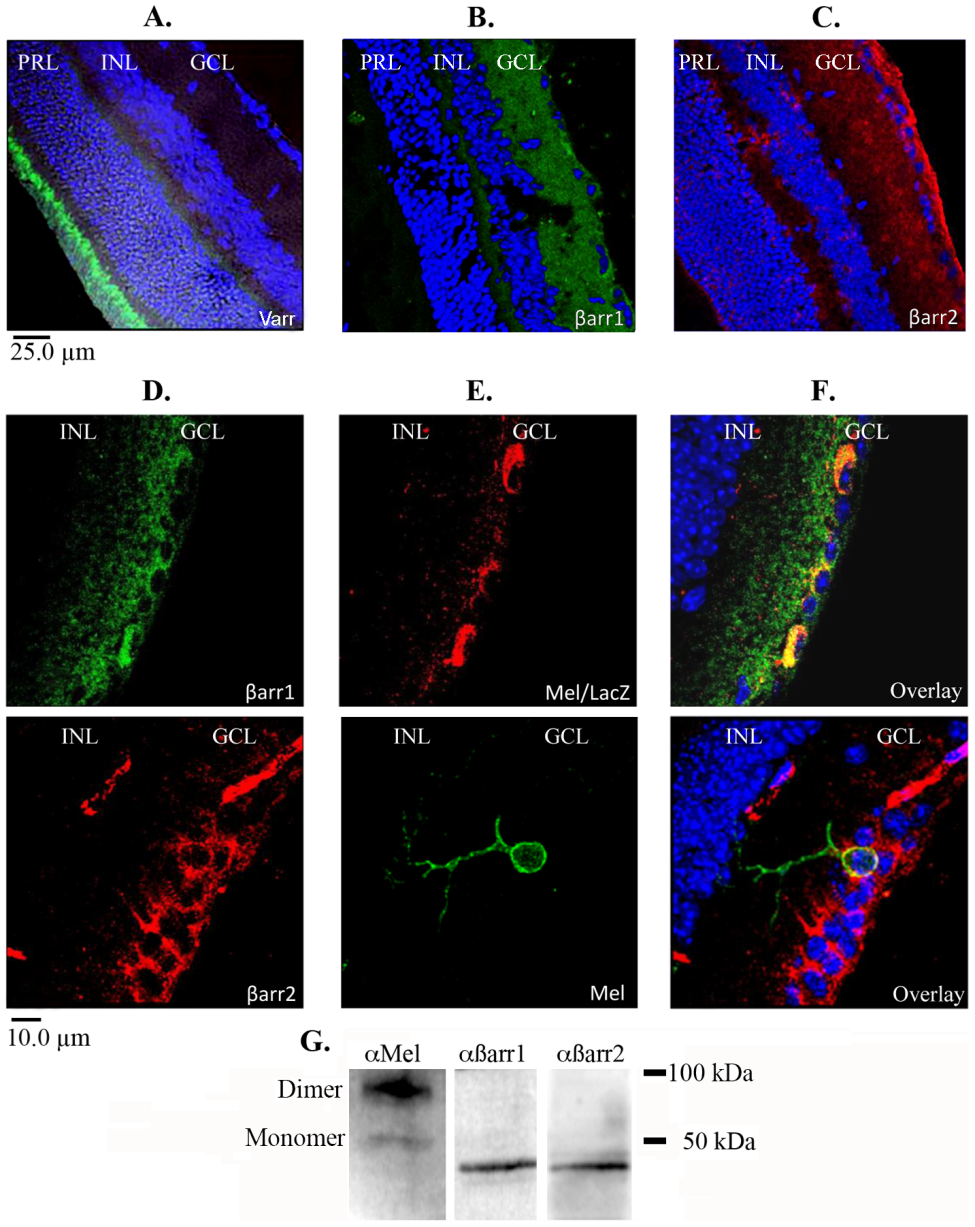
Melanopsin co-localizes with β-arrestin 1 and β-arrestin 2 in ipRGCs. (A–C) Three major nuclear layers of the mouse retina defined by DAPI (GCL, Ganglion cell layer; INL, Inner nuclear layer; PRL, Photoreceptor layer). (A) Visual arrestin staining localized in the PRL outer segments. (B) β-arrestin 1 (β-arr1) and (C) β-arrestin 2 (β-arr2) staining throughout the retina. (D) High magnification of β-arrestin 1 (green) & β-arrestin 2 (red) staining in the GCL cell bodies. (E) Melanopsin expressing cells visualized with anti-β-galactosidase (red) and anti-melanopsin (green) antibodies to prevent cross-reactivity with β-arrestin antibodies during double labeling. (F) Melanopsin co-localizes with β-arrestin 1 and β-arrestin 2 in ipRGC cell bodies. (G) Specificity of melanopsin (N-terminal), β-arrestin 1, and β-arrestin 2 antibodies confirmed by western blot of heterologously expressed protein isolated from HEK293 cells. Melanopsin typically runs as a monomer and dimer with an expected molecular weight of 50 kDa and 100 kDa respectively. β-arrestin 1 and β-arrestin 2 have an expected molecular weight of 48 kDa. 100% of melanopsin expressing cells were positive for β-arrestin 1 (n = 7 cells) and β-arrestin 2 (n = 10 cells) in 14 retinal sections from 4 mice.

Like most GPCRs, visual pigment signaling is terminated in a two-step manner that involves receptor C-tail phosphorylation by G-protein receptor kinases (GRKs) followed by the binding of an arrestin [16], [37]–[39]. It has previously been shown that melanopsin undergoes a light-dependent phosphorylation as part of its deactivation process [35]. In order to determine if arrestin interacts with melanopsin *in vivo*, we examined β-arrestin and visual arrestin expression in mouse retinas by immunohistochemistry. Initially, retinal sections were probed with antibodies raised against visual arrestin, β-arrestin 1, and β-arrestin 2. Not surprisingly, we only observed visual arrestin labeling in the outer segments of the rods and the cones (Fig. 1A). Conversely, β-arrestin 1 and β-arrestin 2 staining localized primarily in ganglion cell layer (GCL) as shown in Figure 1 B–D. We observed the strongest β-arrestin labeling in the GCL cell bodies with sparse β-arrestin labeling in the other layers of the retina. This suggests that the highest concentration of β-arrestin is localized in GCL, but does not rule out β-arrestin expression elsewhere in the retina. It is also possible that the β-arrestin antibodies failed to adequately penetrate the inner layers of the retina resulting in a lack of signal. Melanopsin expressing cells were identified using melanopsin (green) or β-galactosidase (red) specific antibodies (Fig. 1E). Interestingly, the majority of positive melanopsin labeling also localized to the GCL suggesting that melanopsin protein concentrations, like β-arrestin, are highest in the GCL cell bodies. Overlays demonstrate that both β-arrestin 1 and β-arrestin 2 co-localize with melanopsin in ipRGC cell bodies (Fig. 1F), supporting their putative interaction. Of 14 of retinal sections probed, we found that 100% of melanopsin expressing cells were positive for β-arrestin 1 (n = 7 cells) and β-arrestin 2 (n = 10 cells). In the case of β-arrestin 1, double labeling was performed on retinal sections from melanopsin/LacZ (opn4^LacZ/Mel^) transgenic mice which permitted the use of an anti-β-gal antibody to identify melanopsin-expressing cells and prevented secondary antibody cross reactivity.

### Over-expression of β-arrestin increases the rate of melanopsin deactivation, in vitro

**Figure 2:**
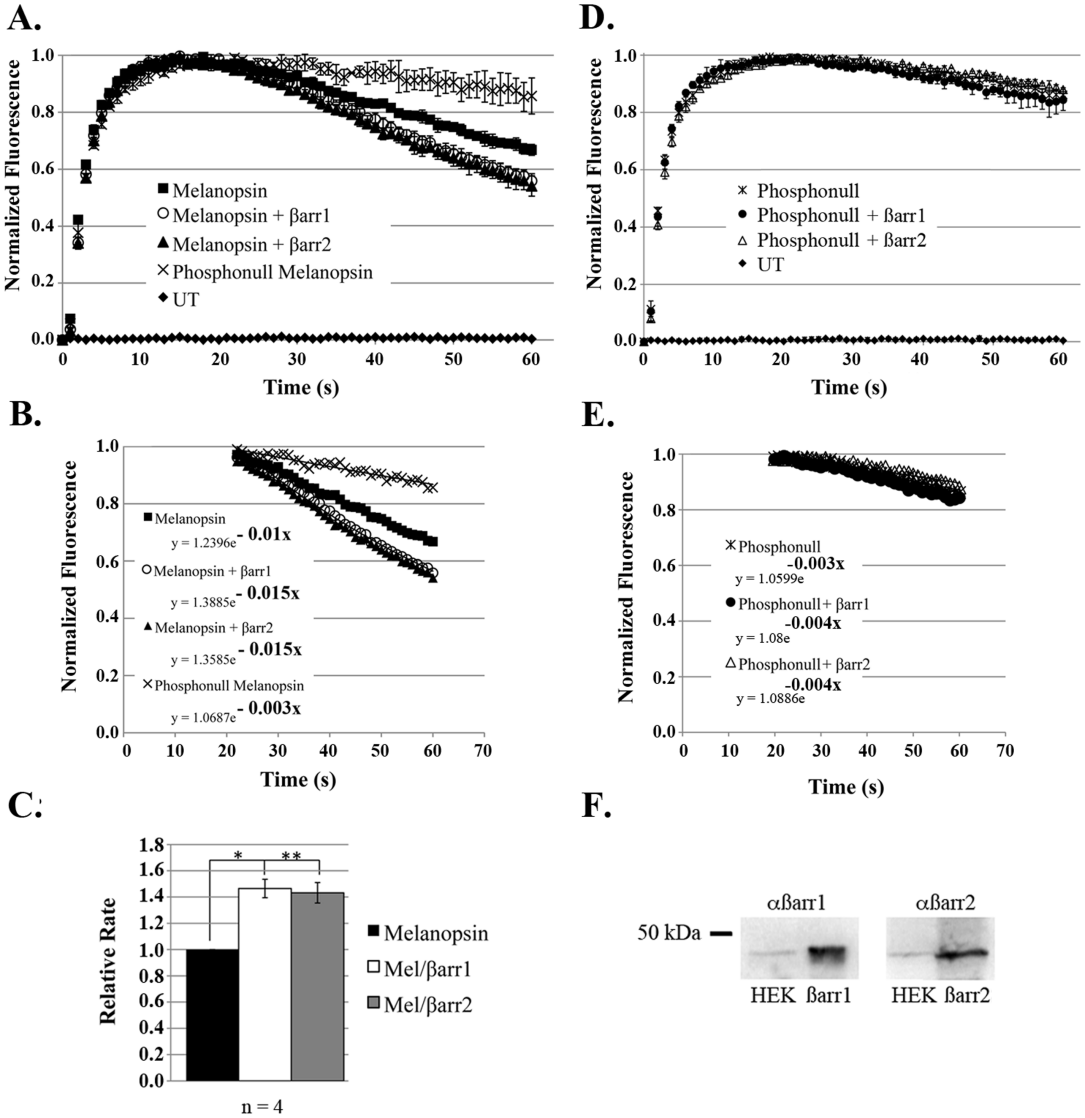
Over-expression of β-arrestin 1 and β-arrestin 2 increases the rate of melanopsin deactivation. (A) Fluorescent calcium imaging responses in HEK293 cells transiently transfected with wildtype melanopsin: wildtype HEK293 (solid squares) and stable cell lines over-expressing either β-arrestin 1 (open circles), or β-arrestin 2 (solid triangles). Increasing the cellular concentration of β-arrestin increases the rate of melanopsin deactivation. The melanopsin mutant lacking C-tail phosphorylation sites (phosphonull) (cross) was also assayed in wildtype HEK293 and deactivates at a slower rate due to the absence of C-tail phosphorylation. (B) The deactivation portion of each kinetic curve was fit to an exponential function and the rate constant for each condition was calculated and is indicated in bold. (C) Comparison of deactivation rate constants for wildtype melanopsin expressed in wildtype HEK293 cells and stable cell lines over-expressing either β-arrestin 1, or β-arrestin 2. This analysis reveals that over-expression of β-arrestin 1 and β-arrestin 2 increases the rate of melanopsin deactivation by 1.5 times. Rate measurements reported as average of 4 independent calcium assays with standard error of the mean reported as error bars. */** denotes statistically significant difference {p<0.05}. (D) Fluorescent calcium imaging responses of the melanopsin mutant lacking C-tail phosphorylation sites (phosphonull) transiently transfected in wildtype HEK293 cells (star) and stable cell lines over-expressing either β-arrestin 1 (solid circle), or β-arrestin 2 (open triangle). (E) Over-expression of β-arrestin does not change the deactivation kinetics of phosphonull melanopsin. An exponential fit of the deactivation portion of these curves reveals little to no difference in the deactivation rate constants indicated in bold. Untransfected cells do not yield a calcium response (solid diamond). All calcium imaging measurements were carried out in triplicate with standard deviation plotted as error bars. Each kinetic curve is representative of 4 independent experiments. (F) Western blot analysis confirming over-expressing β-arrestin 1 and β-arrestin 2 stable cell lines (10–15× higher than endogenous β-arrestin levels).

It has been previously shown that expression of melanopsin in HEK293 cells confers photosensitivity to the cells. Additionally, light activation of melanopsin expressed in HEK293 cells leads to an increase in intracellular calcium that can be recorded as an indirect measure of melanopsin activity [35], [40]. Here we show the melanopsin mediated calcium response in wildtype HEK293 cells and HEK293 cells stably over-expressing β-arrestin 1 or β-arrestin 2. Generally, light-activation of melanopsin expressed in HEK293 cells results in a calcium response that peaks at 15 seconds followed by a declining deactivation phase. When this response was measured in HEK293 cells stably over-expressing β-arrestin 1 or β-arrestin 2, the rate of deactivation was significantly increased (Fig. 2A). In order to directly compare the rates of melanopsin deactivation, we fitted an exponential function to the declining portion of the kinetic curves (Fig. 2B). This generated an equation of exponential decay,
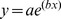
where a = y_max_, e = exponential constant and b = decay coefficient, from which each rate constant (decay coefficient) was compared. Over-expression of β-arrestin 1 and β-arrestin 2 equally increased the rate of melanopsin deactivation compared to endogenous HEK293 β-arrestin levels; both increasing the relative rate of melanopsin deactivation in by 1.5× (Fig. 2C). These results suggest that the concentration of β-arrestin can influence the rate of melanopsin deactivation. Untransfected HEK293 cells yielded no calcium response, and a previously characterized phosphonull melanopsin mutant [35], lacking all C-tail serine and threonines, activated normally but failed to deactivate due to the lack of C-tail phosphorylation (Fig. 2A).

To test whether melanopsin C-tail phosphorylation is a prerequisite for β-arrestin binding, we measured the fluorescent calcium response of the phosphonull melanopsin mutant in HEK293 cells over-expressing β-arrestin 1 or β-arrestin 2. When compared to the phosphonull calcium response measured in wildtype HEK293 cells we found that the deactivation kinetics of the phosphonull mutant was equally delayed regardless of β-arrestin concentration (Fig. 2D). Quantitation of the phosphonull deactivation kinetics further demonstrates this result, and strongly supports a phosphorylation-dependent binding of β-arrestin with melanopsin (Fig. 2E).

### Melanopsin directly interacts with β-arrestin 1 and β-arrestin 2 in a light-dependent manner

**Figure 3:**
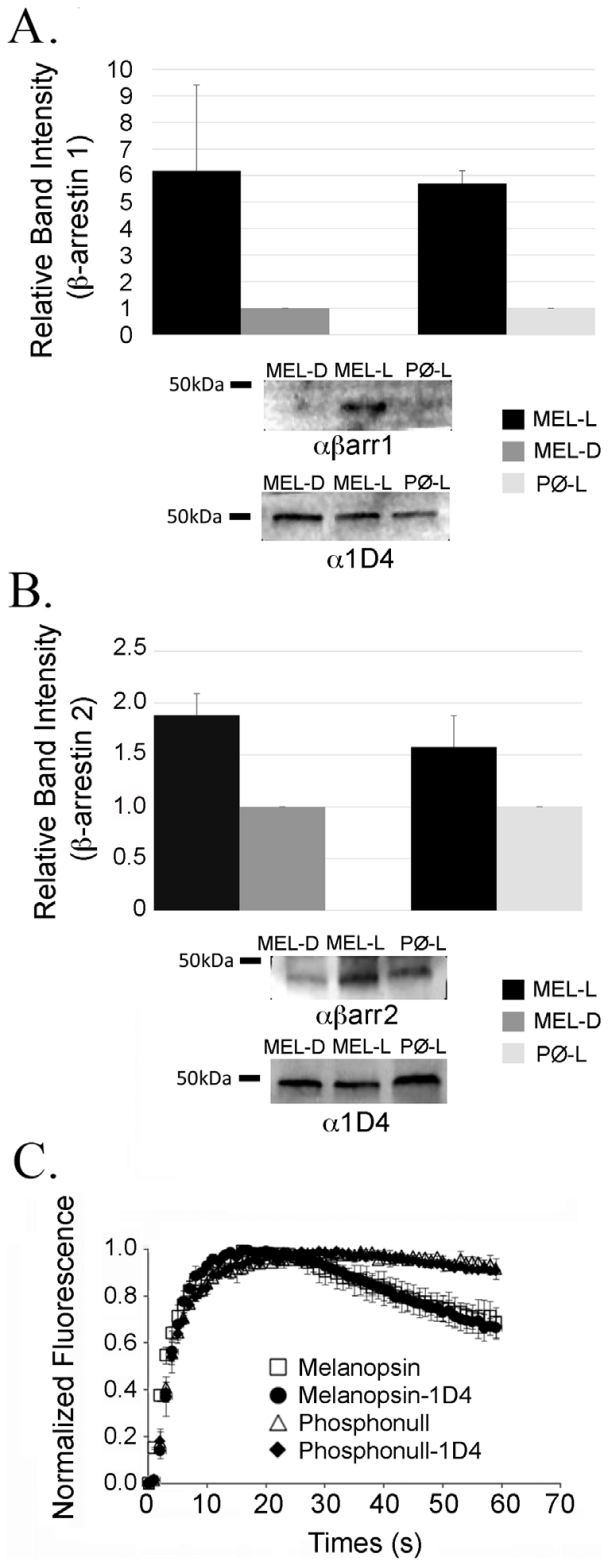
Crosslinking and co-immunoprecipitation of β-arrestin 1 and β-arrestin 2 with melanopsin. β-arrestin 1 and β-arrestin 2 co-precipitate with melanopsin in a light- and phosphorylation-dependent manner. (A) Quantitation of β-arrestin 1 co-precipitation with wildtype (MEL) and phosphonull (PØ) melanopsin samples (top). Binding of β-arrestin 1 to wildtype melanopsin in the light (MEL-L) as compared to the binding to wildtype melanopsin in the dark (MEL-D) and binding of β-arrestin 1 to wildtype melanopsin in the light (MEL-L) as compared to the binding to phosphonull in the light (PØ-L). Representative immunoblot (IB) of β-arrestin 1 co-immunoprecipitation from dark-adapted melanopsin, light-exposed melanopsin and light-exposed phosphonull melanopsin samples (bottom). (B) Quantitation of β-arrestin 2 co-precipitation with wildtype and phosphonull melanopsin samples (top). The relative binding was compared as described above. Representative immunoblot of β-arrestin 2 co-immunoprecipitation from dark-adapted melanopsin, light-exposed melanopsin and light-exposed phosphonull melanopsin samples (bottom). Loading input confirmed by Bradford and anti-1D4 immunobot. Quantitation reported as the average of 4 independent co-immunoprecipitation experiments with standard deviation plotted as error bars. All experiments were carried out in HEK293 cells stably over-expressing β-arrestin 1 or β-arrestin 2 and transiently expressing wildtype melanopsin-1D4 or phosphonull melanopsin-1D4. (C) Addition of the 1D4 epitope does not alter the signaling kinetics of wildtype melanopsin or phosphonull melanopsin in fluorescent calcium imaging responses.

To ascertain whether β-arrestin directly interacts with melanopsin during deactivation we carried out a series of crosslinking and co-immunoprecipitation assays on protein lysates isolated from the over-expressing β-arrestin stable cell lines transiently transfected with wildtype and phosphonull melanopsin. For these experiments, we generated mouse melanopsin and phosphonull melanopsin expression constructs with the 1D4 coding sequence appended to 3′ end. The 1D4 epitope consists of the last 9 amino acids of bovine rhodopsin, providing a simple means of purification with the 1D4 antibody. Addition of the 1D4 epitope to the C-tail of melanopsin did not affect the melanopsin calcium response (Fig. 3C). Using an anti-1D4 immuno-affinity column, melanopsin-1D4 complexes were purified from cell extracts following light exposure and DSP crosslinking [35]. Eluted proteins were separated by SDS-PAGE and probed with anti-β-arrestin 1 and anti-β-arrestin 2 antibodies. We found significantly more β-arrestin 1 and β-arrestin 2 associated with melanopsin in the light-exposed cell lysates compared to dark-adapted samples (Fig. 3). Furthermore, we found a six fold increase in β-arrestin 1 binding to light-exposed wildtype melanopsin as compared to the dark sample and a two fold increase in β-arrestin 2 binding in a similar experiment. When we repeated these experiments with light-exposed phosphonull melanopsin cell lysates, β-arrestin 1 and β-arrestin 2 were detected at comparably lower levels than light-exposed wildtype melanopsin (Fig. 3). Quantitation of β-arrestin 1 and β-arrestin 2 binding to light-exposed wildtype melanopsin as compared to light-exposed phosphonull melanopsin showed a five fold and 1.5 fold increase in binding as a function of phosphorylatable sites, respectively. In the case of the phosphonull/β-arrestin 2 co-precipitation experiment, significantly more β-arrestin 2 was detected than expected. One explanation for this result is that the phosphonull lane was overloaded with protein as suggested by the 1D4 loading control. Furthermore, quantitation of four independent co-immunoprecipitation experiments strongly support a light-dependent melanopsin/β-arrestin interaction that is enhanced by melanopsin C-tail phosphorylation.

**Figure 4:**
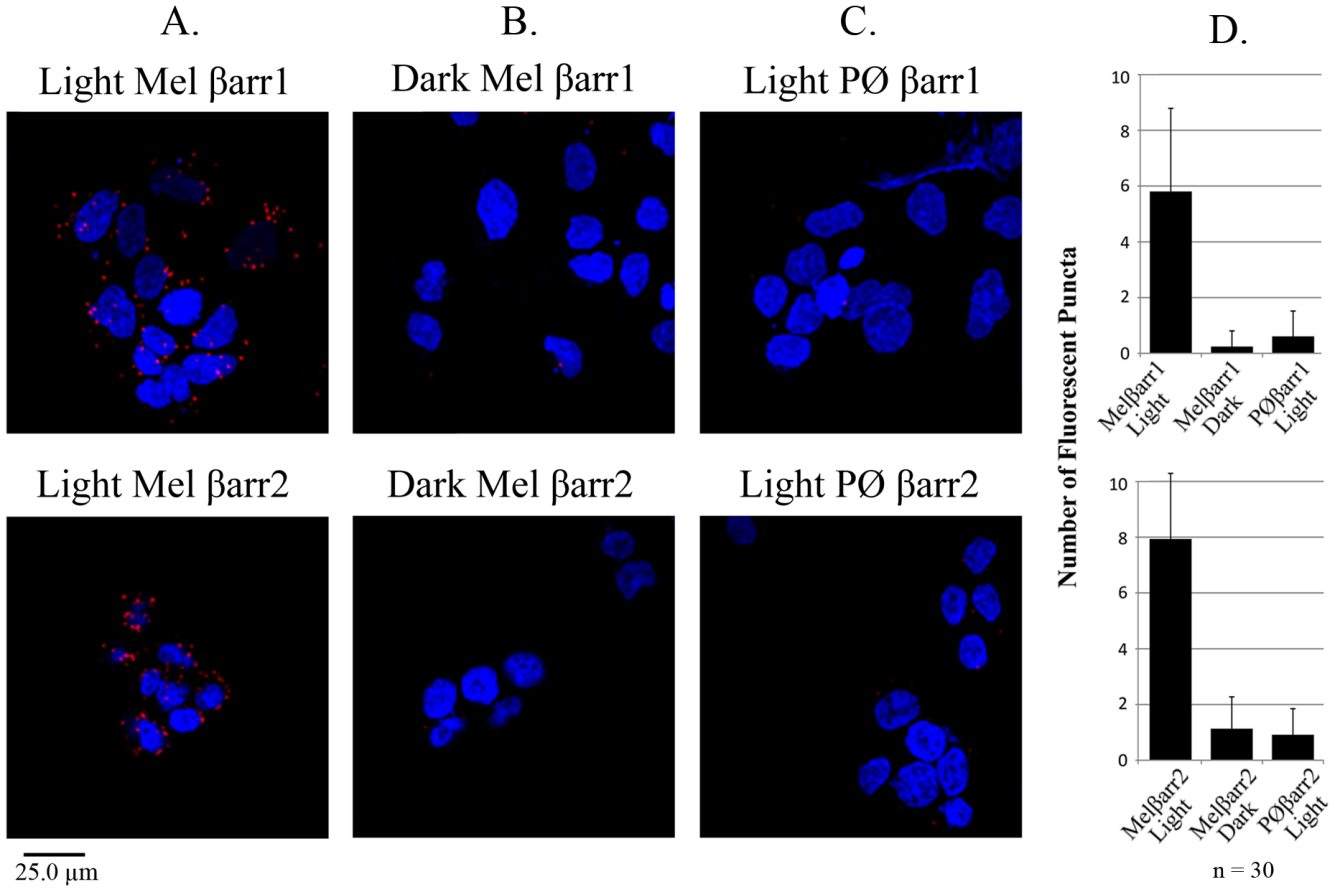
Melanopsin interacts with β-arrestin 1 and β-arrestin 2 in a light- and phosphorylation-dependent manner in fixed HEK293 cells. HEK293 cells heterologously expressing melanopsin and phosphonull melanopsin were assayed by proximity ligation assay (PLA) to investigate the melanopsin/β-arrestin interaction. Cell nuclei labeled with DAPI (blue). Positive PLA interaction indicated by red fluorescent spots. (A) Melanopsin interacts with β-arrestin 1 and β-arrestin 2 in the light, (B) but not in the dark. (C) Phosphonull melanopsin (PØ), lacking C-tail phosphorylation sites, does not interact with either β-arrestin in the light. (D) Histogram showing average number of fluorescent spots per cell in each condition. N = 30 cells. Error bars represent standard error of the mean.

To further demonstrate the melanopsin/β-arrestin interaction, we carried out a series of proximity ligation assays (PLA) on the β-arrestin stable cell lines transiently transfected with melanopsin. The PLA approach allows for the detection of protein-protein interactions within fixed cells or tissues through the use of antibodies. Briefly, antibodies generated against (C-terminal) melanopsin and β-arrestin 1 or β-arrestin 2 were used in conjunction with secondary antibodies conjugated to short oligonucleotides. In a PLA, when two primary antibodies bind in close enough proximity (<40 nm) the secondary antibody oligonucleotides can interact forming a circular piece of DNA that can be amplified and detected with complimentary fluorescent probes [35], [41]. Here we used this approach to confirm the melanopsin/β-arrestin interaction in the light (Fig. 4A) but not in the dark (Fig. 4B). Furthermore, very little PLA signal was detected in light-treated cells expressing the phosphonull melanopsin mutant, supporting a phosphorylation-dependent deactivation mechanism (Fig. 4C). The number of fluorescent puncta was significantly higher (6–8 fold) in the light versus dark and melanopsin versus phosphonull samples (Fig. 4D). These results support the co-immunoprecipitation data (Fig. 3) and imply melanopsin and β-arrestin interact in a light- and phosphorylation-dependent manner HEK293 cells.

### Evidence for a melanopsin/β-arrestin interaction in ipRGCs

**Figure 5:**
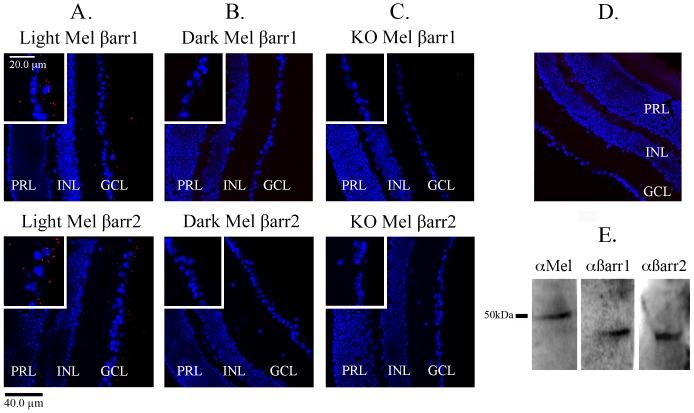
Melanopsin interacts with β-arrestin 1 and β-arrestin 2 in the murine retina. The melanopsin/β-arrestin interaction demonstrated by PLA in mouse retinal sections. Major nuclear layers of the retina defined by DAPI (GCL, Ganglion cell layer; INL, Inner nuclear layer; PRL, Photoreceptor layer). The inserts in A–C represent magnification of the ganglion cell layer. Positive PLA interaction is indicated by red fluorescent spots. (A) A melanopsin/β-arrestin 1 and melanopsin/β-arrestin 2 dependent PLA signal was detected in light-exposed fixed retinas (C57BL/6). (B) Melanopsin does not appear interact with β-arrestin 1 and β-arrestin 2 in dark-adapted fixed retinas. (C) Little PLA signal was detected in light-exposed melanopsin knockout retinas (opn4^LacZ/LacZ^). (D) No signal was detected in retinas (C57BL/6) probed with secondary PLA antibodies only. (E) Specificity of melanopsin (C-terminal), β-arrestin 1, and β-arrestin 2 antibodies confirmed by western blot analysis of protein isolated from mouse retinas.

In order to confirm the melanopsin/β-arrestin interaction in ipRGCs, we performed a series of PLAs on mouse retinal sections. Using light- and dark-adapted retinas isolated from C57BL/6 we detected a melanopsin/β-arrestin-dependent PLA signal (Fig. 5A) in the light, but not in the dark (Fig. 5B). Specifically, we observed a light-dependent PLA signal throughout the entire GCL that extended into the inner plexiform layer (IPL) of the retina. These results suggest that melanopsin interacts with β-arrestin within ipRGC cell bodies and their projections. Unlike our immunohistochemistry results, we observed a positive PLA signal throughout the IPL, supporting a melanopsin/β-arrestin interaction in ipRGC projections. This difference can likely be explained by the signal amplification step intrinsic to the PLA approach that permits detection of proteins/interactions that may not be visible by IHC. As a control we repeated these experiments on light-exposed melanopsin KO retinal sections (opn4^LacZ/LacZ^) and detected very little PLA signal, implying that the interaction is melanopsin dependent (Fig. 5C). Minimal background fluorescence was detected in retinal sections (C57BL/6) probed with secondary antibodies only (Fig. 5D). Western blot analysis of mouse retinal lysates confirms the specificity of the melanopsin and β-arrestin antibodies used in this assay (Fig. 5E).

## Discussion/Conclusion

Here we provide evidence supporting a β-arrestin-dependent deactivation of melanopsin signaling. This result is in contrast with the deactivation of the traditional vertebrate visual pigments expressed in rods and cones, whose signaling is terminated by specialized visual arrestins. Moreover, the melanopsin deactivation mechanism described here differs from that of rhabdomeric visual pigments, which are more closely related to melanopsin. Overall, our data suggest that melanopsin deactivation is more consistent with the canonical two-step deactivation described for other non-visual GPCRs, whereby receptor activation is terminated by C-tail phosphorylation and β-arrestin binding. Generally, GPCR phosphorylation functions to attenuate receptor signaling and also acts to serve as a cue for the activation and binding of a β-arrestin. The subsequent binding of β-arrestin completely quenches GPCR signaling by sterically blocking G-protein interactions with the cytoplasmic loops of the receptor. In many cases, β-arrestin binding leads to internalization of receptor complexes mediated by clathrin and adaptor protein 2 (AP2) binding domains found in β-arrestins' C-tail [14], [15]. This process decreases the number of accessible receptors on the plasma membrane thereby reducing overall sensitivity and expanding the dynamic response range. Interestingly, a variety of GPCR sequence motifs can influence β-arrestins' binding affinity and localization. These determinants can vary from receptor to receptor promoting or inhibiting β-arrestin association [13], [42], [43].

Melanopsin is an unusual visual pigment that is expressed within the retina but outside the rod and cone photoreceptor layer. This therefore raises questions about which arrestin associates with melanopsin during deactivation. Given that melanopsin is expressed within the mammalian retina, it may have seemed likely that it would be deactivated by a visual arrestin. However, the fact that melanopsin expressing retinal ganglion cells (ipRGCs) do not express visual arrestin suggests the possibility that a β-arrestin may quench melanopsin signaling. In order to identify candidate arrestins responsible for melanopsin deactivation, we carried out a series of immunohistochemical labeling experiments on sectioned mouse retinas. Our experiments show that melanopsin co-localizes with both β-arrestin 1 and β-arrestin 2 but not visual arrestin in the GCL. We observed β-arrestin 1 and β-arrestin 2 labeling throughout the entire GCL suggesting that both arrestins are likely co-expressed with melanopsin in all five ipRGC subtypes [23], [44]. These data suggest that *in vivo*, melanopsin interacts with a β-arrestin during deactivation and not visual arrestin. The functional consequences of this interaction were confirmed by calcium imaging, which shows that over-expression of both β-arrestin 1 and β-arrestin 2 increases the rate of the melanopsin deactivation response. The overall effect of β-arrestin over-expression on melanopsin signaling was considerably smaller than expected. This result was likely due to the higher (transient) expression of melanopsin compared to the stably over-expressed β-arrestin. In this instance, a significant number of melanopsin molecules could signal continuously in the absence of β-arrestin binding leading to a smaller increase in the deactivation rate. Nonetheless, β-arrestin over-expression did increase the rate of melanopsin deactivation compared to the melanopsin response in wildtype HEK293 cells, supporting their putative interaction. Future experiments examining the role of stoichiometry on melanopsin signaling could be accomplished by varying protein expression levels and measuring their effect on deactivation. The importance of melanopsin C-tail phosphorylation was demonstrated in calcium imaging experiments testing the effect of increased concentrations of β-arrestin on an unphosphorylated melanopsin protein. These experiments not only highlight the importance of C-tail phosphorylation in melanopsin deactivation but also strongly support C-tail phosphorylation as a prerequisite of β-arrestin binding. To show that melanopsin and β-arrestin directly interact, resulting in deactivation, we carried out co-IP and PLA experiments in transfected HEK293 cells. Both of these experiments independently confirm a light- and phosphorylation-dependent interaction between melanopsin with β-arrestin 1 and β-arrestin 2. Taken together with the retinal IHC data and calcium imaging, we can make a strong argument for a β-arrestin-dependent deactivation of melanopsin. To test for this interaction *in vivo* we repeated the melanopsin/β-arrestin PLA assay in sectioned mouse retinas. These data demonstrate the melanopsin/β-arrestin interaction within fixed retinal ganglion cells, supporting a β-arrestin-dependent deactivation mechanism in the retina. Additionally, published work from our laboratory identified a small set of serine and threonine residues within the proximal portion of the C-tail that are necessary for proper deactivation of melanopsin signaling. Our results support this data and imply that the light-dependent phosphorylation of melanopsin is required for β-arrestin binding in the retina [35].

IpRGCs can be divided into 5 subtypes, each with different signaling kinetics and response sensitivities [23], [44]. Determining the level of melanopsin and β-arrestin protein expression within each ipRGC subtype may explain these diverse response characteristics. Experiments focused on determining which β-arrestin preferentially binds melanopsin during deactivation should also provide insight into how regulation of melanopsin signaling influences the cellular response. Future work should also be aimed at understanding how other regions within melanopsin's C-tail contribute to signaling and deactivation. Compared to rhodopsin and cone opsins, melanopsin has an extremely long C-tail with over 120 additional amino acids, 34 of which are predicted to be phosphorylated by various kinases including GRKs, protein kinases, and tyrosine kinases [45]. Investigating the phosphorylation states of these predicted sites may lead to a greater understanding about how melanopsin signaling is regulated.

Overall, this work strongly supports a β-arrestin-dependent deactivation of melanopsin signaling. Together, our *in vitro* and *in situ* data support this mechanism within melanopsin expressing retinal ganglion cells. Additionally, the phosphorylation-dependent interaction between β-arrestin and melanopsin is consistent with canonical deactivation mechanism described for other GPCRs.
